# The recommendations of Chinese Parkinson’s disease and movement disorder society consensus on therapeutic management of Parkinson’s disease

**DOI:** 10.1186/s40035-016-0059-z

**Published:** 2016-06-30

**Authors:** Shengdi Chen, Piu Chan, Shenggang Sun, Haibo Chen, Baorong Zhang, Weidong Le, Chunfeng Liu, Guoguang Peng, Beisha Tang, Lijuan Wang, Yan Cheng, Ming Shao, Zhenguo Liu, Zhenfu Wang, Xiaochun Chen, Mingwei Wang, Xinhua Wan, Huifang Shang, Yiming Liu, Pingyi Xu, Jian Wang, Tao Feng, Xianwen Chen, Xingyue Hu, Anmu Xie, Qin Xiao

**Affiliations:** Department of Neurology, Ruijin Hospital affiliated to Shanghai Jiao Tong University School of Medicine, Shanghai, 200025 China; Xuanwu Hospital affiliated to Capital Medical University, Beijing, China; Tongji Hospital affiliated to Tongji Medical College of Huazhong University of Science and Technology, Wuhan, China; Beijing Hospital, Beijing, China; The Second Hospital affiliated to Zhejiang University School of Medicine, Hangzhou, China; The First Hospital affiliated to Dalian Medical University, Dalian, China; The Second Affiliated Hospital of Soochow University, Suzhou, China; The First Hospital affiliated to Chongqing Medical University, Chongqing, China; Xiangya Hospital, Central South University, Changsha, China; Guangdong General Hospital, Guangzhou, China; General Hospital affiliated to Tianjin Medical University, Tianjin, China; Sichuan Rehabilitation Hospital, Chengdu, China; Xinhua Hospital affiliated to Shanghai Jiaotong University School of Medicine, Shanghai, China; Chinese PLA General Hospital, Beijing, China; Fujian Medical University Union Hospital, Fuzhou, China; The First Hospital affiliated to Hebei Medical University, Shijiazhuang, China; Peking Union Medical College Hospital, Beijing, China; West China Hospital affiliated to Sichuan University, Chengdu, China; Qilu Hospital affiliated to Shandong University, Jinan, China; The First Hospital affiliated to Guangzhou Medical University, Guangzhou, China; Huashan Hospital affiliated to Fudan University, Shanghai, China; Tiantan Hospital affiliated to Capital Medical University, Beijing, China; The First Hospital affiliated to Anhui Medical University, Hefei, China; Sir Run Run Shaw Hospital affiliated to Zhejiang University School of Medicine, Hangzhou, China; The Affiliated Hospital, Qingdao University School of Medicine, Qingdao, China

**Keywords:** Parkinson’s disease, Treatment guideline, optimal therapeutic options, China

## Abstract

**Background:**

Parkinson’s disease (PD) is a chronic, progressive and debilitating disease, which affects over 2.5 million people in China. PD is characterized clinically by resting tremor, muscular rigidity, bradykinesia and postural instability. As the disease progresses, additional complications can arise such as non-motor and neurobehavioral symptoms. Pharmacological treatment and surgical intervention for PD have been implemented in China. Until 10 years ago, there was lack of standardization for the management of PD in different regions and among different physicians, leading to different treatment levels in different regions and different physicians. Since then, the Chinese Parkinson’s Disease and Movement Disorder Society have published three versions of guidelines for the management of PD in China, in 2006, 2009 and 2014, respectively. Correspondingly, the overall level of treatment for PD in China improved.

**Objectives:**

To update the treatment guidelines based on current foreign and domestic practice guidelines and clinical evidence, and to improve the treatment options available to physicians in the management of PD.

**Summary:**

A variety of treatment recommendations in the treatment guidelines have been proposed, including physical activity and disease-modifying medication, which should be initiated at the early-stage of the disease. The principles of dosage titration should be followed to avoid acute adverse reactions to the drugs, to achieve a satisfactory clinical effect with a low dose and to reduce the incidence of long-term motor complications. Moreover, different treatment strategies should be considered at different stages of the disease. Importantly, treatment guidelines and personalized treatments should be valued equally. A set of treatment recommendations has been developed to assist physicians to improve and optimize clinical outcomes for patients with PD in China.

## Background

Parkinson’s disease (PD) is the second most common age-dependent neurodegenerative disorder, affecting approximately 1.7 % of the population over 65 years old in China [1, 2]. A variety of environmental, genetic, immunological cues, including excessive oxidative stress, have been proposed to be associated with the onset of this disease [3]. The defining neuropathological features of PD are the preferential loss of dopaminergic (DA) neurons and the formation of Lewy bodies in the substantia nigra pars compacta (SNpc) [3]. However, the molecular mechanism underlying neurodegeneration in PD is not fully understood. The clinical manifestations of PD include motor symptoms, e.g., resting tremor, muscular rigidity, bradykinesia, and postural instability; and non-motor symptoms, e.g., hyposmia, rapid eye movement (REM) sleep behavior disorder, constipation and depression [3–6].

The motor symptoms of PD can be alleviated by drugs that enhance dopamine function, including levodopa, dopamine agonists, type B monoamine oxidase (MAO-B) inhibitors, catechol O-methyl transferase (COMT) inhibitors, amantadine and benzhexol. Among these, levodopa is considered the most effective. However, chronic use of these drugs leads to loss of their therapeutic efficacies and motor complications. In addition, some of non-motor symptoms of PD cannot be improved significantly and comprehensively using these drugs.

Motor and non-motor symptoms markedly affect the quality of life of PD patients. Optimized treatment is essential to improve symptoms and enhancing the quality of life of PD patients. During the past 10 years, we have developed three treatment guidelines for PD patients in China in 2006, 2009, and 2014, respectively [7–9]. The implementation of these guidelines has promoted greatly the standardization and optimization of the management of PD patients in China. Here, we provide updated treatment guidelines to assist physicians to improve and optimize clinical outcomes for patients with PD in China.

## Contents

### Principles of therapy

#### Comprehensive treatment

PD patients can present sequentially or simultaneously with either motor or non-motor symptoms. However, these symptoms could be present in the patients throughout their treatment and affect the quality of the patient’s life. Therefore, comprehensive treatment for both motor and non-motor symptoms should be implemented. The recommended therapeutic strategies include medication, surgical intervention, physical activity and other rehabilitation, psychological counseling, and caring and nursing assistance. Medication is recommended as the first option for PD treatment, and is considered the key approach throughout the course of treatment, whereas surgery is suggested as an effective supplement to medication. However, the currently used therapeutic strategies can only improve the symptoms of PD patients, without modifying or curing the disease. Therefore, the management of PD should focus not only on immediate management, but also on long-term management, to obtain long-term benefits for patients.

#### Principles of medication

Both motor and non-motor symptoms can affect the working ability and quality of life in PD patients; therefore, it is recommended that the principles of medication should aim at effectively improving symptoms, working ability and quality of life. Physical activity and disease-modifying medication should be initiated at the early-stage of the disease, which could not only alleviate the symptoms, but also delay the progression of the disease. The principles of dosage titration should be followed to avoid acute adverse reactions to the drugs, achieve a satisfactory clinical effect with a low dose and reduce the incidence of motor complications. Interestingly, the incidence of dyskinesia in Chinese PD patients is significantly lower than that in Western countries [10–12].

In addition, the treatment of PD should be based on evidence-based medicine; however, personalized medicine is advocated. In other words, to make a decision on a medication, a comprehensive assessment of the patient is required, including the dominant symptoms, disease severity, as well as a variety of factors, such as cognitive condition, age of onset of the disease, employment status, comorbidities, adverse effects of the drugs, the patients preferred drug selection and economic status. Every effort should be made to avoid, delay and reduce the acute side effects, as well as the chronic motor complications. Anti-PD drugs, particularly levodopa, should not be discontinued abruptly to avoid the occurrence of the symptom aggravation or malignant withdrawal syndrome.

### Pharmaceutical therapies

PD can be classified according to disease severity, into the early stage (Hoehn − Yahr: 1 − 2.5) and the advanced stage (Hoehn − Yahr: 3 − 5). Therapeutic options are suggested for the treatment of early stage and advanced stage of PD, independently.

#### Treatment of early-stage PD

PD progresses gradually with time. Clinical evidence indicates that PD progresses faster in the early stage than in the advanced stage. Therefore, once diagnosed in the early stage, treatment, particularly disease-modifying medication or physical activity, is required as early as possible to make optimally use of the best time window in PD progression. The treatment of early stage PD should be divided into drug treatment and non-drug treatment (including physical activity, adding knowledge of the disease, providing nutritional supplementation, increasing building up confidence regarding conquering the disease, and gaining support from family and society). At the early stage, monotherapy is usually considered; however, combined therapy in minimal doses can also be adopted to achieve the goals with the best therapeutic effect, and to maintain a long-term impact to reduce the incidence of motor complications as much as possible.

Medication includes using disease-modifying drugs and symptom-treating drugs. In addition to the capacity for disease modification, disease-modifying drugs can also improve symptoms, while some symptom-treating drugs might also have disease-modifying effects.

The purpose of using disease-modifying drugs is to delay disease progression. Clinically, the most commonly used disease-modifying drugs include monoamine oxidase-B (MAO-B) inhibitors and dopamine agonists. Clinical trials indicated that an MAO-B inhibitor (selegiline) combined with tocopherol (the DATATOP trial) and rasagiline (the ADAGIO trial) could delay the progression of PD [13, 14]. Furthermore, it was suggested that dopamine agonists, such as pramipexole (the CALM-PD trial) and ropinirole (the REAL-PET trial), might have disease-modifying effects [15, 16]. However, there is insufficient evidence to show that these drugs have disease-modifying effects.

##### Principles of drug selection

1.  Recommendations for the management of early-onset PD patients without cognitive impairment (Fig. 1) include: ① non-ergot dopamine agonists; ② MAO-B inhibitors alone, or together with tocopherol; ③ Amantadine; ④ levodopa plus decarboxylase inhibitors (levodopa/benserazide or levodopa/carbidopa); ⑤ Stalevo (levodopa/carbidopa + COMT inhibitor); and ⑥ anticholinergics (benzhexol).

**Fig. 1:**
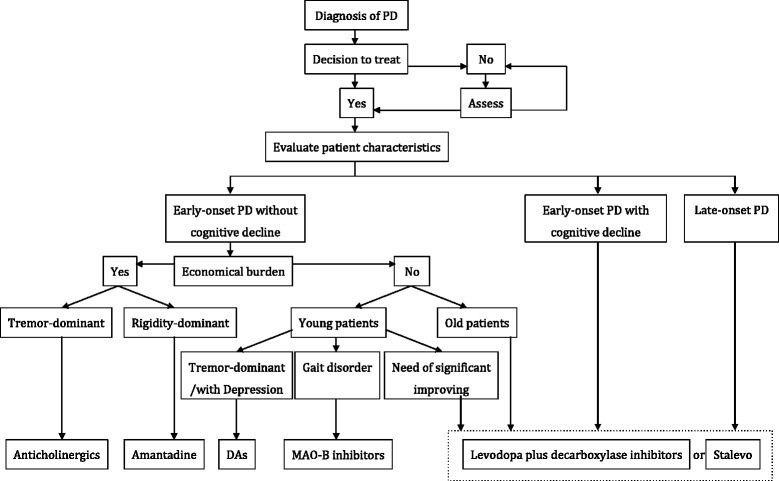
Pathway for monotherapy in early Parkinson’s disease
  The principles of drug selection are not dependent on the sequence of the above recommendations; instead, the choice should be only based on the particular condition of each PD patient. It is recommended to start with non-ergot dopamine agonists, MAO-B inhibitors, or Stalevo, for early-onset young patients. Dopamine agonists, especially pramipexole, are recommended as preferable for tremor-dominant patients or PD patients with depression, while MAO-B inhibitors are recommended as preferable for PD patients with gait disorders. Amantadine can be initially used if it is economically infeasible to start with expensive drugs. For patients with special occupations, or for the purpose of significantly improving motor symptoms, or upon cognitive decline, it is recommended to start with levodopa plus decarboxylase inhibitors, or Stalevo. Additionally, the minimal dose of dopamine agonists, MAO-B inhibitors, or amantadine can be co-applied with a minimal dose of levodopa plus decarboxylase inhibitors. For patients with severe tremor-dominant symptoms, anticholinergics (such as benzhexol) could be used when other anti-PD drugs are not effective; however, the related side effects must be closely monitored.
2.  Recommendations for the management of late-onset PD patients or early-onset PD patients with cognitive impairment. Levodopa plus decarboxylase inhibitors are recommended as the first choice medication. However, with the progression of the disease, dopamine agonists, MAO-B inhibitors, or COMT inhibitors could be considered as add-on therapies (Fig. 2). Benzhexol is not recommended because of possible severe adverse effects, particularly for older male patients.

**Fig. 2:**
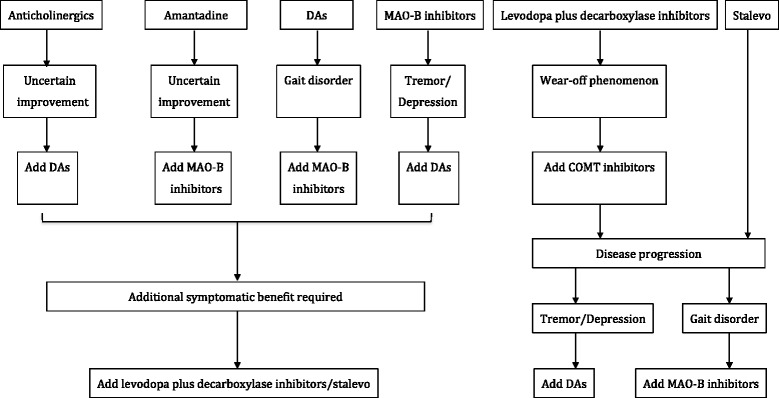
Pathway for combined therapy in early Parkinson’s disease

##### Therapeutic drugs options

1.  Levodopa plus decarboxylase inhibitors. The recommended initial dosage is 62.5 − 125 mg two times or three times per day (b.i.d, or t.i.d) and the dosage needs to be increased gradually as the disease progresses, until symptoms improve, or the appropriate dosage sustained until adverse effects are absent. The drug should be taken one hour before meals or one and half hour after meals. In the past, a delay in the use of levodopa was recommended, because dyskinesia could be induced by early use of levodopa. However, the principle of early used low dose had been proposed for many years in China and several studies provided evidence that early use of a low dose (≤400 mg/d) of levodopa did not increase the incidence of dyskinesia [10, 11, 17], which was supported by the results of Olanow’s study [12]. Immediate-release levodopa/benserazide or levodopa/carbidopa shows fast acting effects, while controlled-release levodopa/benserazide or levodopa/carbidopa produces slow acting effects with low bioavailability. Therefore, patients should be careful when taking the two different types of drugs. Furthermore, they should be used with caution in patients with active peptic ulcers, and should be avoided in patients with narrow cleft glaucoma or mental disorders. The dispersible formulation of levodopa/benserazide is easy to use and shows rapid therapeutic efficacy after ingestion. Thus, it is recommended for PD patients with morning akinesia, delayed onset of “on” time, afternoon “off” status, and dysphagia [18].
2.  Dopamine agonists. Non-ergot dopamine agonists are the most commonly used first-choice drugs [19–24], and are particularly suitable for early-stage PD patients. Their long half-lives mean that such drugs could avoid pulse stimulation on the striatal postsynaptic membrane, thereby probably preventing or decreasing the likelihood of motor complications. Dopamine agonists should be started at lower doses, and then increased slowly until the dosage is reached that shows both a satisfactory effect and the absence of adverse effects. The adverse reactions to dopamine agonists are similar to those found with levodopa. However, dopamine agonists have a lower incidence of motor fluctuation and dyskinesia and a higher incidence of orthostatic hypotension, ankle edema, and mental disorders (illusion, bulimia and hypersexuality). There are two types of dopamine agonists: ergot dopamine agonists (bromocriptine, pergolide, dihydroergocryptine, cabergoline and lisuride) and non-ergot dopamine agonists (pramipexole, ropinirole, piribedil, rotigotine and apomorphine). Ergot dopamine agonists are currently not recommended because of their potential severe adverse reactions, such as valvular disorder and fibrosis of the lung pleura [25–27]. Thus, pergolide has already been discontinued in China. These severe adverse reactions to pergolide were rarely found in China, because of lower dosage used compared with Western countries. Currently, the most commonly used non-ergot dopamine agonists recommended in China are pramipexole and sustained-release piribedil. They have been used for over 10 years. Ropinirole is now available for use in the clinic. A multi-center clinical trial of rotigotine has been completed and it will soon be available for clinical use.
  ①  Pramipexole: There are two types of formulations: immediate-release pramipexole and extended-release pramipexole. For immediate-release pramipexole, the initial dosage is 0.125 mg, t.i.d.; this should then be increased by 0.125 mg, t.i.d., on a weekly basis. The usual clinically effective dose is 1.5 − 2.25 mg/d, up to a maximum dosage of 4.5 mg/day; however, very few Chinese patients take this dosage [24]. A randomized, double-blind, double-dummy, parallel-group study of the efficacy and safety of extended-release pramipexole versus immediate-release pramipexole conducted in Chinese PD patients showed similar efficacy and safety profiles. Immediate-release pramipexole can be switched to extended-release pramipexole overnight or gradually over a few days at the same daily dose [24, 28].
  ②  Sustained-release piribedil: The initial dosage is 50 mg q.d or 25 mg b.i.d. during the first week, which is then increased to 50 mg, b.i.d., from the second week on. The usual clinically effective dose is 50 mg, t.i.d., and the maximal dosage is 250 mg/day; however, very few Chinese patients take this dose.
    The forthcoming non-ergot dopamine agonists recommended in China are ropinirole and rotigotine.
  ③  Ropinirole: The initial dosage is 0.25 mg, t.i.d., which can be increased each week until the dosage reaches 1.0 mg, t.i.d. The usual clinically effective dose is 1.0 − 3.0 mg, t.i.d., and the maximal daily dosage (24 mg/day), which has been used in a few Chinese patients [29].
  ④  Rotigotine: The initial dosage should be 2 mg, one time per day (q.d.), which may be increased by 2 mg weekly. The usual clinically effective dose is 6 − 8 mg/day for early-stage patients and 8 − 16 mg for advanced-stage patients [30].
    The ergot dopamine agonists, including bromocriptine and α-Dihydroergocryptine, are currently available in China but they are now not recommended in Chinese PD patients.
3.  MAO-B inhibitors. These include selegiline and rasagiline. They are recommended for the treatment of both early stage and advanced stage PD, particularly in the early onset or de novo patients. An initial dosage of selegiline is 2.5–5 mg, b.i.d., which should be taken in the morning and at noon, but not in the evening to avoid insomnia, or can be taken together with tocopherol (2,000 IU) [13]. The recommended dosage of rasagiline is 1.0 mg, q.d. after breakfast [31, 32]. These should be used with caution in patients with gastric ulcers, and taking them together with 5-hydroxytryptamine-selective serotonin reuptake inhibitors should be avoided, because of possible severe adverse reactions [33, 34].
4.  COMT inhibitors. One of the main causes of motor complications is the “pulse” - like stimulation of the striatal postsynaptic dopamine receptor. COMT inhibitors produce sustained dopaminergic stimulation on striatal postsynaptic dopamine receptor. The combination of entacapone/levodopa/carbidopa, i.e., Stalevo, is recommended for the early stage of PD to improve symptoms and to prevent or delay the onset of motor complications [35]. However, it is controversial whether early-stage use of Stalevo can delay the onset of motor complications or increase the incidence of dyskinesia; this awaits further validation [36]. For advanced stage patients, entacapone or tolcapone can be added as an adjunct therapy when the therapeutic effects of levodopa/benseraside or levodopa/carbidopa decline [17]. The dosage of entacapone is 100 − 200 mg/time, and the frequency of administration is similar to that of levodopa/benseraside or levodopa/carbidopa [36, 37]. It is recommended that entacapone be taken together with levodopa/benseraside or levodopa/carbidopa. The dosage of tolcapone is 100 mg, t.i.d., and the first dosage should be taken together with levodopa/benseraside or levodopa/carbidopa and should be repeated every 6 h, or it can be used alone, at a maximum dosage of 600 mg per day [38]. The adverse reactions to COMT inhibitors include diarrhea, headache, sweating, dry mouth, increased alanine aminotransferase, abdominal pain and discoloured urinecolor yellow. Tolcapone could lead to liver damage; therefore, liver function should be monitored closely, particularly in the first 3 months of use.
5.  Amantadine. It is recommended to start amantadine at a dosage of 50 − 100 mg b.i.d.; the patient should take the last dose before 4:00 PM. Amantadine can improve hypokinesia, rigidity and tremor, and can also improve dyskinesia (Level C evidence). Caution should be taken in using amantadine in PD patients with renal insufficiency, epilepsy, severe ulcers and liver disease. It should never be taken by lactating women.
6.  Anticholinergics. Benzhexol (1 − 2 mg, t.i.d.) is the most commonly used anticholinergic drug in China, mainly in early-onset young patients, but not in elderly patients. It is recommended that this be applied only in young patients with tremor-dominant symptoms, usually when other anti-PD drugs are not effective. For patients aged < 60 years, patients should be informed that long-term use of benzhexol could result in cognitive decline; thus, regular examination of cognitive function is required. Once cognitive decline is confirmed, the drug should be discontinued promptly. Usually, benzhexol is not recommended for PD patients aged ≥ 60 years. Its use is contraindicated for patients with narrow angle glaucoma or prostatic hypertrophy.

#### Treatment of advanced-stage PD

The treatment of advanced-stage PD is extremely complicated, involving the progression of the disease, the decline of therapeutic effects, and adverse reactions or motor complications after chronic use of medication [39, 40]. To treat patients with advanced PD, it is recommended that physicians aim to improve motor symptoms and manage motor complications and non-motor symptoms.

#### Management of motor complications

Motor complications, including motor and non-motor symptom fluctuations and dyskinesias, are presented frequently in advanced-stage PD [41, 42]. Adjusting the dosage or frequency of medication can improve symptom fluctuations or dyskinesias. Moreover, surgical intervention, such as deep brain stimulation (DBS), can also be effective [20, 35, 43–46].

#### Management of symptom fluctuations

The common symptom fluctuations include the wearing-off and the on − off phenomena. It is recommended that the wearing-off phenomenon can be managed using several approaches (Table 1).

**Table 1:** Recommendation for the management of the wearing-off phenomenon

① Increase the frequency of drug taking with the same daily dose, or appropriately increase the total daily dose. ② Switch immediate-release levodopa to controlled-release levodopa or extended-release levodopa to prolong the action of levodopa. They are more appropriate for the wearing-off phenomenon in early-stage PD. The dosage of controlled-release levodopa should be increased by 20 − 30 % after switching from immediate-release levodopa, because of its low bioavailability. The US guidelines reported that this could not decrease the off stage (Level C evidence) [44], while the UK NICE guidelines recommended this application in advanced-stage PD, but not as the first choice (Level B evidence) [92]. ③ Add long half-life dopamine agonists, including pramipexole and ropinirole (Level B evidence), cabergoline and apomorphine (Level C evidence) [44]; however, bromocriptine could not shorten the off stage (Level C evidence) [44]. Other dopamine agonists could be used to replace the currently used dopamine agonists that lose their efficacy. ④ Add COMT inhibitors to generate continuous dopaminergic stimulation to the striatum, including entacapone (Level A evidence) and tolcapone (Level B evidence) [44]. ⑤ Add MAO-B inhibitors, such as rasagiline (Level A evidence) and selegiline (Level C evidence) [44]. ⑥ Minimize the impact of protein diet on the uptake and blood − brain barrier crossing of levodopa. Drugs should be taken 1 h before or 1.5 h after meals. ⑦ Surgical intervention, such as DBS of the subthalamic nucleus (STN) (Level C evidence), is helpful.	

The management of the on − off phenomenon is very complicated. It is recommended that treatment is started with long half-life dopamine agonists orally, or continuous micropump infusion of levodopa methyl ester, ethyl ester, or dopamine agonists (such as lisuride) [19, 35, 43–45, 47].

#### Management of dyskinesias

Dyskinesia includes peak-dose dyskinesia, biphasic dyskinesia, and dystonia. Several approaches are recommended to manage peak-dose dyskinesia (Table 2).

**Table 2:** Recommendation for the management of peak-dose dyskinesia

① Reduce the single dose of levodopa/benseraside or levodopa/carbidopa per time; or appropriately add dopamine agonists or COMT inhibitors if the motor symptoms deteriorate after the dose of levodopa is reduced. ② Add amantadine (Level C evidence) [44]. ③ Add atypical neuroleptics, such as clozapine, but start with an initial low dosage and then increase gradually, and closely monitor granulocytes. ④ Replace controlled-release levodopa with immediate-release levodopa to avoid the cumulative effects of controlled-release levodopa, which can aggravate dyskinesia.	

Several approaches are recommended to manage biphasic dyskinesias (Table 3).

**Table 3:** Recommendation for the management of biphasic dyskinesias

① Replace controlled-release levodopa with immediate-release levodopa, preferably with madopar dispersible, which can be used to manage the beginning-of-dose dyskinesia. ② Add long half-life dopamine agonists or COMT inhibitors, which can extend the half-life of plasma levodopa, thus increasing the area under the curve, and relieving the end-of-dose dyskinesia. This may also benefit the beginning-of-dose dyskinesia.	

Continuous micropump infusion of dopamine agonists, levodopa methyl or ethyl ester can improve both dyskinesias and symptom fluctuations. However, it is still under investigation whether oral preparations can achieve similar effects. Other drugs for the treatment of dyskinesias, including adenosine A2A receptor antagonists, which target non-DA receptors in the basal ganglia, are currently in clinical trials.

For the management of morning dystonia, it is recommended to add controlled-release levodopa other than immediate-release levodopa, or long-acting dopamine agonists at night, and take an immediate-release levodopa other than controlled-release levodopa, especially madopar dispersible, before getting up in the morning. The management of on-dystonia is similar to that of peak-dose dyskinesia. Surgical intervention, such as DBS, can be beneficial to manage dyskinesias [48–52].

#### Management of postural instability

Postural instability is the most common cause of falling in advanced-stage PD patients, which occurs easily during postural changes such as turning around, getting up or bending down. Currently there are no effective therapeutic approaches, although adjusting the dosage or adding other drugs may occasionally help. In the initial stages, it may be beneficial to adjust the center of gravity (by stepping, striding and walking), to listen to commands, music or beating rhythms, or to stride across objects [53]. When necessary, effective supervision and care, as well as the use of walking aids or wheelchairs, are needed.

#### Management of non-motor symptoms

The non-motor symptoms are very common clinical manifestations in advanced-stage PD patients. They include sensory disorders, mental disorders, autonomic dysfunction and sleep disorders, which require active management and treatment [54, 55].

① *Management of mental disorders*. The most common mental disorders in advanced-stage PD are depression and/or anxiety, illusion, cognitive disorders and dementia. First, it should be identified whether anti-PD drugs induce the symptoms or whether they are caused by the disease itself. For drug-induced symptoms of mental disorders, it is recommended that the drugs be reduced or discontinued, based on risk in the following order: anticholinergics, amantadine, MAO-B inhibitors and dopamine agonists. If the symptoms persist after taking the above measures, it may be necessary to reduce the dosage of levodopa/benseraside or levodopa/carbidopa gradually without aggravating the PD motor symptoms. If no satisfactory response is gained after adjusting the dosage, this would indicate that the disease itself might be causing the symptoms. Therefore, the drug therapy for mental disorders should be considered. For patients with hallucinations and illusions, clozapine or quetiapine is recommended. Clozapine has a slightly stronger effect than quetiapine, but there is a slight likelihood of agranulocytosis (1–2 %). Thus, close monitoring of the blood count is necessary. The use of antipsychotics (APs) is associated with an increased risk of mortality in patients with PD; quetiapine was associated with the lowest (but still elevated) risk [56]. Pimavanserin, a selective inverse agonist of the 5-hydroxytryptamine 2A (5-HT_2A_) receptor, has been approved by the United States Food and Drug Administration [57]. Data from phase II and phase III clinical trials suggest that pimavanserin is a safe and effective treatment option for psychosis in PD patients [58, 59]. Trial results indicate a significant reduction in hallucinations and delusions in PD patients with psychosis, without worsening motor symptoms [58, 59]. For the treatment of depression and/or anxiety, it is recommended that dopamine agonists, especially pramipexole, which can both improve the motor symptoms and depression, or selective serotonin reuptake inhibitors, be used. Lorazepam and diazepam are highly effective for irritability. Cholinesterase inhibitors, such as rivastigmine and donepezil, and memantine are recommended to treat cognitive disorders and dementia. Rivastigmine is recommended as the first choice [60–62].
② *Management of autonomic dysfunction*. The most common symptoms of autonomic dysfunction include constipation, urinary dysfunction and orthostatic hypotension. To improve constipation, adequate intake of fluid, fruits, vegetables, fiber and lactulose (10–20 g/day) or other mild cathartics, Chinese traditional medicine, (including rhubarb, aloe, long hui pill and folium sennae) might be helpful. Adding gastric dynamic drugs, such as domperidone or mosapride, might also be beneficial. It is recommended that anticholinergics be discontinued if they are used together with other anti-parkinsonian drugs. For the treatment of frequent micturition, urgent micturition and urge incontinence, peripheral anticholinergics, including oxybutynin, propantheline, tolterodine and hyoscyamine, are recommended. Cholinergics are recommended for patients with detrusor areflexia. However, these drugs should be used with caution, as they can aggravate the motor symptoms of PD. If urinary retention is present, intermittent catheterization should be considered. If the urinary retention is caused by prostate hypertrophy, surgical intervention should be considered. Patients with orthostatic hypotension need to increase their intake of salt and water, to raise the head of their bed during sleep and to avoid rising too quickly from a lying or sitting position. Elastic leg stockings may also be useful. Midodrine, a highly effective α-adrenergic agonist, is recommended as the first-choice drug [60, 63, 64]. Alternatively, domperidone, a selective peripheral dopamine antagonist, could be considered [65–67].
③ *Management of sleep disorders*. Sleep disorders are very common symptoms in PD patients. They include insomnia, REM behavior disorder (RBD) and excessive daytime sleepiness (EDS). Difficulty in maintaining sleep (sleep fragmentation) is the most common manifestation of insomnia. Frequent waking may lead to the appearance of tremors during the light-stage of sleep. Patients may be unable to move, resulting in difficulties in turning over or increased nocturia, which may be caused by the loss of the dopaminergic action. Supplementation of controlled-release levodopa, dopamine agonists, or COMT inhibitors before bedtime might be helpful if it is associated with PD symptoms at night. For patients taking selegiline or amantadine in the evening, the medication time should be adjusted, i.e. selegiline should be taken in the morning and at noon, and amantadine should be taken at least before 16:00. If the sleep disorders do not improve, reducing the dosage or even discontinuing those drugs should be considered. Alternatively, short-acting hypnotics or sedatives could be recommended. For patients with RBD, clonazepam can be taken at bedtime. EDS may be related to the severity of PD and cognitive decline, and can also be associated with the use of dopamine agonists and levodopa. If sleepiness occurs after taking medication, it may suggest overdosing; therefore, reducing the dose may help to improve EDS. Three RCTs have assessed the efficacy of modafinil to treat EDS in PD. Based on these studies, which had conflicting efficacy results [68–70], there is insufficient evidence tocome to a conclusion on the efficacy of modafinil for the treatment of EDS in PD [65]. Only one study examined the effects of methylphenidate (30 mg qd) for the treatment of EDS or fatigue in PD in a double-blind, placebo-controlled parallel-group RCT [71]. Although results appear to be positive, there is insufficient evidence to conclude that methylphenidate is safe and efficacious for the treatment of EDS or fatigue in PD [65]. Substituting immediate-release levodopa with controlled-release levodopa might help to avoid or reduce sleepiness after medication.
④ *Management of sensory disturbances*. The most common sensory disturbances occurring in PD patients are hyposmia, pain or numbness, and restless legs syndrome (RLS) [6, 72]. Hyposmia is likely to occur a number of years before the appearance of motor symptoms [73, 74]. However, there are no effective measures to improve it. Pain or numbness is particularly common in patients with advanced PD, and can be caused by the disease itself, or by accompanied bone and joint lesions. If pain or numbness can be relieved during the on-period of medication, but recur during the off-period, it might be caused by PD; thus, the long half-life dopamine agonists or COMT inhibitors should be recommended to prolong the on-period. Alternatively, if it results from another cause, relevant symptomatic therapies should be implemented. For PD patients with RLS, taking a dopamine agonist, e.g. pramipexole or ropinirole, 2 h before bedtime, is highly effective; levodopa is also efficacious. Opioid drugs might be considered for RLS refractory to dopaminergic drugs.

### Surgical treatment

Surgical treatment should be considered when the motor symptoms are not significantly improved and severe motor fluctuations or dyskinesias occur. Nucleus lesion and deep brain stimulation (DBS) are the main surgical approaches for PD. DBS is the main choice because of its non-invasive, safe and controllable advantages [44]. The indications for DBS intervention for PD include: (1) Idiopathic PD with more than 5 years and Hoehn-Yahr 2.5–4; (2) age below 75 years old; (3) a good response to levodopa/benseraside or levodopa/carbidopa at the early stage of the disease (levodopa challenging test shows more than 30 % improvement of motor symptoms evaluated by UPDRS-III, although the efficacy of medication is declining) [75–77]; (4) severe motor fluctuations or dyskinesias occur, which affects the quality of the patient’s life; and (5) dementia and severe mental illness must be excluded [78]. It should be emphasized that DBS can only improve the motor symptoms significantly, but does not cure the disease. Therefore, postoperative medication is still required,; however, the dosage can be reduced. DBS shows good therapeutic efficacy for limb tremor and/or muscular rigidity, but no significant effect in PD patients with postural instability. DBS targets for PD include the subthalamic nucleus (STN) and globus pallidus internus (GPi), and could significantly improve tremor, muscular rigidity, bradykinesia and dyskinesias. A good preoperative response to levodopa could serve as prognosis indicator for patients who have undergone DBS of the STN (Level B evidence) [44], while young patients with a short course of the disease show better improvement than older patients with a long course (Level C evidence) [44]. However, there is no evidence for an association between GPi or VIM and prognosis after DBS (Level U evidence) [44].

### Exercise and other complementary management

Exercise and other complementary management strategies, including Tai chi, Qi gong, yoga, massage, acupuncture, dance and traditional herbs have shown efficacy in improving the motor symptoms and even possibly delaying disease progression [79–81]. They could be helpful to PD patients with gait disorders, postural instability, language disorders and dysphagia. In addition, persistent training for language disorder, gait disorder and postural instability might also help to improve the capacity for self-care and motor function, and could prolong the duration of the efficacy of medication [82–86].

### Psychological counseling

Psychological disorders, such as anxiety and depression, are common in PD patients. Anxiety and, particularly, depression are important risk factors that affect the quality of patient life and the effectiveness of PD medication [87–91]. They can occur before and after the onset of the motor symptoms of PD. Therefore, medication should not only focus on improving the motor symptoms, but also on depression. Psychological counseling and anti-depressants should be recommended to achieve more satisfactory improvements.

### Caring and nursing

Reasonable care and nursing assistances should be used as adjunct treatment for controlling disease status and for improving symptoms, in addition to professional treatment of PD patients with medication. For example, changing the diet composition and training swallowing skills may be helpful for PD patients with dysphagia; and using a straight strap by deputy chair and not using too deep, too soft a chair or sofa are beneficial for PD patients with difficulty in sitting and standing. These measures could also avoid effectively accidents, such as aspiration events and falling.

## Conclusions

Parkinson’s disease is a highly complicated and incurable disease, particularly in its advanced stage. In clinical practice, guidelines for the management of PD are helpful to achieve optimal treatment for PD patients. Moreover, it is important to determine the status of dominant symptoms and disease severity, how patients respond to medication, including efficacy, on and off time and adverse reactions, before making a treatment decision. As different patients may have different dominant symptoms and different sensitivities to treatment, they will have different medical requirements. In addition, the same patient requires different treatment adjustments in different stages of the disease; therefore, there are no fixed patterns for the management of PD patients. Importantly, it should be emphasized that the optimized therapeutic efficacy can be achieved only if personalized therapy and the therapeutic guidelines are adopted.

## Abbreviations

COMT: Catechol O-methyl transferase
DA: Dopaminergic
DBS: Deep brain stimulation
EDS: Excessive daytime sleepiness
GPi: Globus pallidus internus
MAO-B: Type B monoamine oxidase
PD: Parkinson’s disease
RBD: REM behavior disorder
REM: Rapid eye movement
SNpc: Substantia nigra compacta
STN: Subthalamic nucleus
